# The Phylogenetic Characterization of *Balantioides coli* Isolated in the Pavlova Culture Medium Supplemented with Coconut Water and Animal Serum

**DOI:** 10.3390/pathogens13060476

**Published:** 2024-06-04

**Authors:** Camila Souza Carvalho Class, Laís Lisboa Corrêa, Fabiana Batalha Knackfuss, Maria Regina Reis Amendoeira, Francisco Ponce Gordo, Alynne da Silva Barbosa

**Affiliations:** 1 Microbiology and Parasitology Department, Biomedical Institute, Federal Fluminense University, Professor Hernani de Mello Street, 101, São Domingos, Niterói 24210-130, RJ, Brazil; camilaclass@id.uff.br (C.S.C.C.); laislisboa@id.uff.br (L.L.C.); 2 Zootecnia e Estatística, Universidade do Grande Rio, Professor José de Souza Herdy Street, 1160, Jardim Vinte e Cinco de Agosto, Duque de Caxias 25071-202, RJ, Brazil; fbknackfuss@hotmail.com; 3 Protozoology Laboratory, Oswaldo Cruz Institut, Oswaldo Cruz Foudation, Brazil Avenue, 4365, Manguinhos, Rio de Janeiro 21045-900, RJ, Brazil; amendoeira.fiocruz@gmail.com; 4 Departamento de Parasitología, Facultat de Farmacia, Universidad Complutense de Madrid, Plaza Ramón y Cajal, 28040 Madrid, Spain; pponce@farm.ucm.es

**Keywords:** molecular characterization, in vitro culture, *Balantioides coli*

## Abstract

*Balantioides coli* is a ciliated protist that can cause dysentery in humans, pigs and nonhuman primates and may have the potential for zoonotic transmission. Its diagnosis is routinely performed through conventional parasitological techniques, and few studies have used culturing techniques to isolate it, applying molecular tools for the characterization of this protozoan. Thus, the objective of this study was to confirm *B. coli* diagnosis using molecular tools and to characterize the genetic variants of this parasite isolated from pigs kept on family farms in Brazil using three different culture media that differed in the serum added. Fecal samples from pigs were inoculated in Pavlova medium plus coconut water (PC), fetal bovine serum (PB) and horse serum (PH). Of the 127 samples positive for forms compatible with the phylum Ciliophora, 31 were selected for isolation. The most successful medium for isolation was PB 19/31 (61.3%), followed by PH 18/31 (58.1%) and PC 11/31 (35.5%). Of the nucleotide sequences generated, 20 were classified as genetic variant type B0, two as A1 and 15 as A0. The results indicated that PC, despite having allowed the isolation of *B. coli* for a short period, was not an adequate medium for the maintenance of this parasite in vitro, therefore requiring improvement.

## 1. Introduction

*Balantioides coli* is a ciliated protist that can infect several animal species, including pigs and nonhuman primates, and may affect zoonotic transmission cycles [1,2,3]. Balantiosis is a highly neglected parasitic disease that can be acquired indirectly by ingesting cyst-contaminated food and water [1,2,4]. This parasite inhabits the large intestine and can cause several clinical manifestations in infected hosts, especially severe dysentery and extraintestinal colonization [4,5].

The diagnosis of *Balantioides coli* has been performed with microscopic parasitological examinations for the detection of trophozoites and cysts in biological samples. However, due to the limitations of these microscopic techniques, molecular tools have been increasingly used in studies of ciliated protists to accurately identify the parasite species and to describe the genetic variants of these agents [6,7]. For *B. coli*, few molecular markers have been described, and the main target used has been ribosomal RNA, especially the region ITS1.5.8S.ITS2. Through analyzing the ITS regions, *B. coli* isolates can now be classified into A0, A1, A2, B0 and B1 [8].

In vitro culture is another research tool that can be used for studies of intestinal protists [9]. Among the advantages of performing such cultures is that a significant number of parasitic cells can be used for different purposes, such as in the study of metabolism; immunodiagnostic, morphological, molecular epidemiology and drug evaluation and experimental infection [10,11,12]. In contrast, to perform the isolation and maintenance of these microorganisms in vitro culture systems, equipment, reagents, and other supplies are required that are not always available, making the process expensive and laborious [12]. The culture media used for this purpose are complex and usually composed of different reagents that act as sources of peptides, amino acids, nucleic acids, carbohydrates, lipids and vitamins [10]. Among these inputs, whey stands out as a source of nutrients rich in lipids that ensure the growth of parasitic cells in vitro. Generally, the sera used are of animal origin and include fetal bovine, horse and human sera [10].

To make in vitro cultivation less expensive and more accessible by reducing costs, the use of alternative methodologies to replace the use of serum from an animal origin is necessary. Coconut water is a natural solution that contains a variety of nutrients, including minerals, proteins, sugars, vitamins, growth factors and other nutrients [13]. In addition, coconut water is a low-cost organic component and is, therefore, a strong candidate for replacing serum of animal origin [14]. However, few studies have added this nutrient source to in vitro culture media to isolate and maintain different microorganisms [15,16,17,18]. Among the articles in the literature to date, coconut water has not yet been tested for ciliated protists.

Therefore, this study aimed to confirm *B. coli* diagnosis using molecular tools and to characterize genetic variants of this parasite isolated from pigs kept on family farm facilities in Rio de Janeiro, Brazil, using three different culture media that differed in the type of serum added (fetal bovine serum, horse serum and coconut water). 

## 2. Materials and Methods

### 2.1. Collection of Fecal Samples from Family Pig Farms

This study was conducted between December 2020 and August 2021 using fecal samples from pigs kept on family farms located in the municipality of Cachoeiras de Macacu, Rio de Janeiro. Fecal samples from 180 pigs were collected from the rectal ampulla with a long palpation sleeve. Collections were performed on 10 properties, which are referred to by letter codes to preserve their anonymity. On farm A, there were 36 pigs; on B, 3; on C, 6; on D, 27; on E, 8; on F, 4; on G, 15; on H, 28; on I, 41 and on J, 12. Although no clinical analysis was performed on the animals, at the time of collection, the majority of feces were neither diarrheal nor dysenteric. It is worth noting that family farms, as they do not have sophisticated technical standards, have variable numbers of animals, so feces were collected from all animals present on them, excluding only lactating piglets and pregnant sows.

### 2.2. Selection of Samples and In Vitro Isolation of Ciliated Protozoa

After direct examination, the positive samples, i.e., those in which cysts and trophozoites compatible with *Balantioides coli* were identified and selected for in vitro isolation. The samples selected had at least 10 mobile trophozoites and/or 30 cysts on direct examination according to a previously standardized protocol [19].

The inoculum was prepared from fresh feces eluted in a sterile buffered saline solution. The inoculation of the samples was always performed in duplicate using two tubes containing 8 mL of the three different culture media (PC, PB and PH). A total of 180 µL of fecal solution was added to each tube. Soon after, the material was incubated vertically in a microbiological oven at 36 °C.

Before the inoculation of the selected material, as well as after each maintenance subculture, 60 µL of sterile rice starch suspension was added to test tubes containing fresh medium.

After 24 h of incubation, 60 µL of the sediment was removed, and the sediment was subsequently deposited on a microscope slide covered with a 24 × 32 mm coverslip. The slides were read using a light microscope at 100× magnification (Primo Star, Zeiss^®^, Oberkochen, Germany). In cultures where it was possible to visualize mobile trophozoites, 180 µL of the sediment was transferred to a tube containing fresh medium. The negative tubes, that is, those without moving trophozoites, were reincubated for another 24 h and subsequently checked. In these cases, the incubation and verification procedures were performed three times, for a maximum period of 72 h; at the end of this period, if the tubes remained negative, the material was discarded. Isolation was performed when there were mobile trophozoites in this verification stage. The isolates that were successfully maintained until the 5th day of incubation in 24 h subcultures were considered to be in maintenance. Thereafter, the cells were subcultured in fresh media at 48 to 72 h intervals.

### 2.3. Preparation of the Culture Medium

Three culture media that differed in serum composition, namely, PC, PB and PH, were used in this study. Coconut water (PC), fetal bovine serum (PB) and horse serum (PH) were added to the autoclaved xenic medium of Pavlova modified with Jones [20,21]. In the preparation of the modified Pavlova complete medium, 50 mL of the respective sera was added to each 1 L of sterile base medium.

Fetal bovine serum was purchased commercially (Cultilab^®^, Campinas, SP, Brazil), and horse serum was acquired through a donation and partnership with Vital Brazil Institute. Coconuts were purchased commercially from a supermarket on the same day that the base medium was prepared to ensure that the coconut water was as fresh as possible. All the manipulations were performed in a laminar flow hood to maintain a sterile environment. In the laboratory, the external part of the coconut was sanitized with 70% alcohol. Subsequently, the water was transferred to a sterile container, and the solution was immediately added to the base medium. The gelatinous portion of the fruit was discarded. The coconut water was not autoclaved to prevent the degradation of the organic components by heating.

In the assembly of the medium, antibiotic solutions composed of streptomycin sulfate at a final concentration of 500 µg/mL and penicillin solution at a final concentration of 1000 µL/mL were also added. Finally, 8 mL of the complete medium was poured into sterile glass test tubes (20 mm × 150 mm) with screw-top lids.

### 2.4. DNA Extraction from Cultured Samples, Polymerase Chain Reaction and DNA Purification

All the samples isolated in culture that remained viable until the 5th day of incubation were subjected to molecular characterization. The High Pure PCR Template Preparation Kit from Roche (Basel, Switzerland) was used according to the manufacturer’s recommendations to extract DNA from the samples in cultivation. After all the steps were performed, the extracted DNA was stored in a freezer.

The polymerase chain reaction (PCR) was performed with 45 µL of Supermix (Invitrogen^®^, Itapevi, SP, Brazil), 8 µL of the extracted DNA, 1 µL of the forward primer B58D (5′ GCTCCTACCGATACCGGGT 3′) and 1 µL of the reverse primer B58RC (5′ GCGGGTCATCTTACTTGATTTC 3′), which amplifies the ITS1–5.8 s rRNA–ITS2 region of ciliated protozoa as previously recommended [22]. The PCR was performed in an automatic thermocycler (Veriti 96-Well Thermal Cycler, Thermo Fisher Scientific, Waltham, MA, USA). The final product of the 500 bp reaction was visualized on a 1.5% agarose gel using Gel Red, which was observed with a transilluminator.

The amplicon purification was performed using an Illustra™ Kit (GFX™ PCR DNA and Gel Band Purification Kit, Global Life Sciences, Amersham, UK). The purified DNA was also subjected to visualization on a 1.5% agarose gel with Gel Red. Finally, the purified material was subjected to DNA sequencing on the Fiocruz Genomics Platform using an Applied Biosystems 3730xl 96-capillary sequencer (model ABI 3500xL). The sequences were obtained from both directions (forward and reverse) with the same primers used in the PCR.

The analysis and initial editing of the sequences were performed using Chromas Pro software, version 1.7.5. Subsequently, the BLASTn analysis tool was used to compare the data obtained with reference sequences belonging to the same gene fragment stored in the GenBank database. The sequences were saved in Fasta mode and aligned with other homologous sequences taken from the GenBank database using BioEdit software, version 7.2.5. Phylogenetic inferences were obtained from maximum likelihood analyses for confirmation with bootstrap selection and 1000 replications. The best evolutionary model was selected based on the Akaike information criterion (AIC) using W-IQ-Tree software (http://iqtree.cibiv.univie.ac.at/, accessed on 29 April 2022). Phylogenetic tree editing and rooting were performed using MEGA-X software, version 11. Sequences selected from the *B. coli* GenBank dataset and their genetic variants (A0, A1, A2, B0 and B1) detected from different hosts were included as references and reference sequences from other ciliate species were used to form the outgroups. These included morphologically similar protozoa such as *Buxtonella* sp., *Buxtonella sulcata* and *Balantidium entozoon* [6,23]. In addition, phylogenetically distant ciliated protozoa such as *Troglodytella abrassarti* and *Isotricha prostoma* were used for the rooting of the phylogenetic tree [24,25].

### 2.5. Analysis of Results

The isolation and maintenance of ciliated protists in each culture medium were assessed, and the descriptive results are presented according to the maximum period of viability of the isolates, that is, via the visualization of mobile cells under microscopy. The number of viable parasite isolates in the in vitro culture media was calculated based on the time, and the significance of the frequency of viability of the isolates was compared between the three media (PC, PB and PH) using the chi-square test. The analysis was carried out in SPSS (Statistical Package for the Social Sciences), version 29.0.2.0, at the 5% significance level. In addition, the photodocumentation of the culture material visualized under light microscopy was also performed through the 5th day of maintenance, the incubation time at which the isolates underwent molecular characterization.

## 3. Results

Of the 127 fecal samples positive for evolutionary forms morphologically compatible with *B. coli*, 31 were selected for in vitro isolation, i.e., at least 10 mobile trophozoites and/or 30 cysts were observed in the direct examination. Of these, ciliated protist isolates were obtained from 19 samples and were successfully maintained in vitro until the 5th day of incubation in an oven, i.e., up to 120 h in at least one of the culture media used (Table 1). In addition, at least one successfully maintained isolate was obtained from each family pig farm included in this study (Table 1).

All the selected samples that followed the isolation pattern for trophozoites (at least 10 mobile trophozoites on direct examination during the selection sample step), that is, six samples were successfully maintained through the 5th day in at least one of the culture media. The selection pattern with the detection of cysts (at least 30 cysts in direct examination) was evidenced in 18 samples; however, in only 6 samples were isolates of the protozoan obtained. The isolation of the protozoan from both evolutionary forms was observed in 10 samples, with successful isolation in 7 of those samples (Table 1).

By visualizing the parasites moving in the culture media, it was observed that successful isolation, i.e., the maintenance of isolates until the 5th day of incubation, occurred mainly with modified Pavlova medium plus fetal bovine serum (PB) 19/31 (61.3%), followed by Pavlova modified with horse serum (PH) 18/31 (58.1%) and, finally, Pavlova modified with coconut water (PC) 11/31 (35.5%), as highlighted in the gray part of Table 2. The long-term maintenance of the ciliated protist, i.e., cultivation that exceeded one month, was only achieved in the PB and PH media (Table 2). In general, the majority of isolates obtained in PC were only maintained for up to 24 h (up to the 1st day of incubation). However, parasite death in PB and PH culture media also occurred in this time range, highlighting the lack of statistical significance (*p* > 0.05) of the frequency of parasite viability among the three different media within the 1st day of incubation. The only period for which there was a statistical difference (*p* ≤ 0.05) among the three different media was the interval of 1 to 2 days of incubation (Table 2). Two isolates from samples 44 and 53 were maintained in vitro for a long period, i.e., more than one year, with 44 being maintained in PH and 53 in PB.

**Figure 1:**
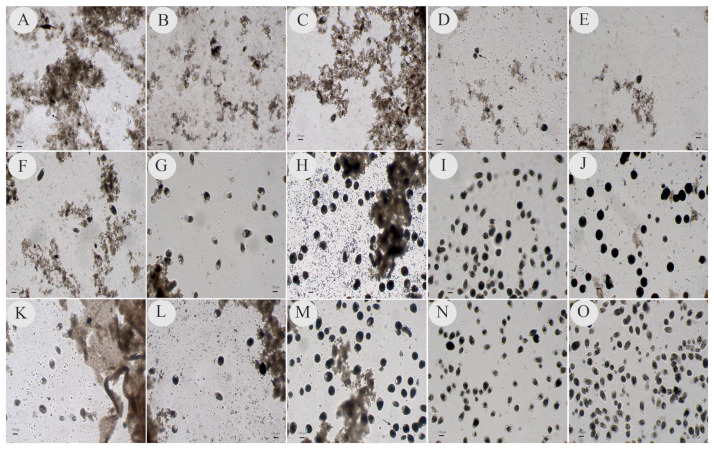
Photos reflecting samples collected after 24 h (1st day), 48 h (2nd day), 72 h (3rd day), 96 h (4th day) and 120 h (5th day) of incubation in an oven for the isolation of ciliated protozoans in modified Pavlova plus coconut water (PC), modified Pavlova plus fetal bovine serum (PB) and modified Pavlova plus horse serum (PH). Photos (**A**–**E**) correspond to the isolates in the PC medium; photos (**F**–**J**) correspond to the isolates in the PB medium and photos (**K**–**O**) correspond to the isolates in the PH medium. Photos (**A**,**F**,**K**) show the 24 h incubation duration; photos (**B**,**G**,**L**) show 48 h; photos (**C**,**H**,**M**) show 72 h; photos (**D**,**I**,**N**) show 96 h and photos (**E**,**J**,**O**) show 120 h. Divisions are highlighted by the tips of the arrows. The photos were taken at 100× magnification; the scale bar represents 150 µm.

In general, the tubes containing the material inoculated for up to 72 h (3rd day) had many fecal remnants, and debris could be visualized on the microscope slides when checking for isolates. However, the ciliates showed faster movement and more translucent cytoplasmic staining after up to 48 h (2nd day), but especially after 24 h (1st day), of incubation than at the other times (Figure 1A,B,F,G,K,L). From 72 h onwards, the movement speed began to decrease, and the transverse division of the parasitic cells intensified in all media from which the isolates were successfully obtained (Figure 1C,H,M). In the subsequent subcultures at 96 and 120 h (4th and 5th days), the ciliate forms were rounder, larger, darker, and less motile than they were at the initial times, and little evidence of fecal material was observed on the microscope slides (Figure 1D,E,I,J,N,O). During these periods, large numbers of parasites were observed on microscope slides during the observations and production of subcultures, mainly in PB and PH (Figure 1I,J,N). The evolutionary forms of other microorganisms, such as bacteria, parabasilids and *Blastocystis* sp., were also visualized during the observations.

Of the 19 samples in which in vitro isolates of the ciliated protozoan were successfully obtained and that were molecularly characterized, 10 samples generated amplified products in the PCR and came from two culture media that differed in the type of serum added. In five samples, the DNA was amplified via PCR for isolates generated from only one type of culture medium, and in only four samples, the product generated via PCR was obtained from ciliates isolated in Pavlova medium plus the three types of serum (Table 1).

Most of the ciliated protozoan DNA amplicons that were subjected to genetic sequencing were derived from isolates of the parasite in Pavlova medium plus fetal bovine serum (17 isolates), followed by Pavlova medium plus horse serum (14 isolates) and, finally, Pavlova medium plus coconut water (6 isolates).

**Figure 2:**
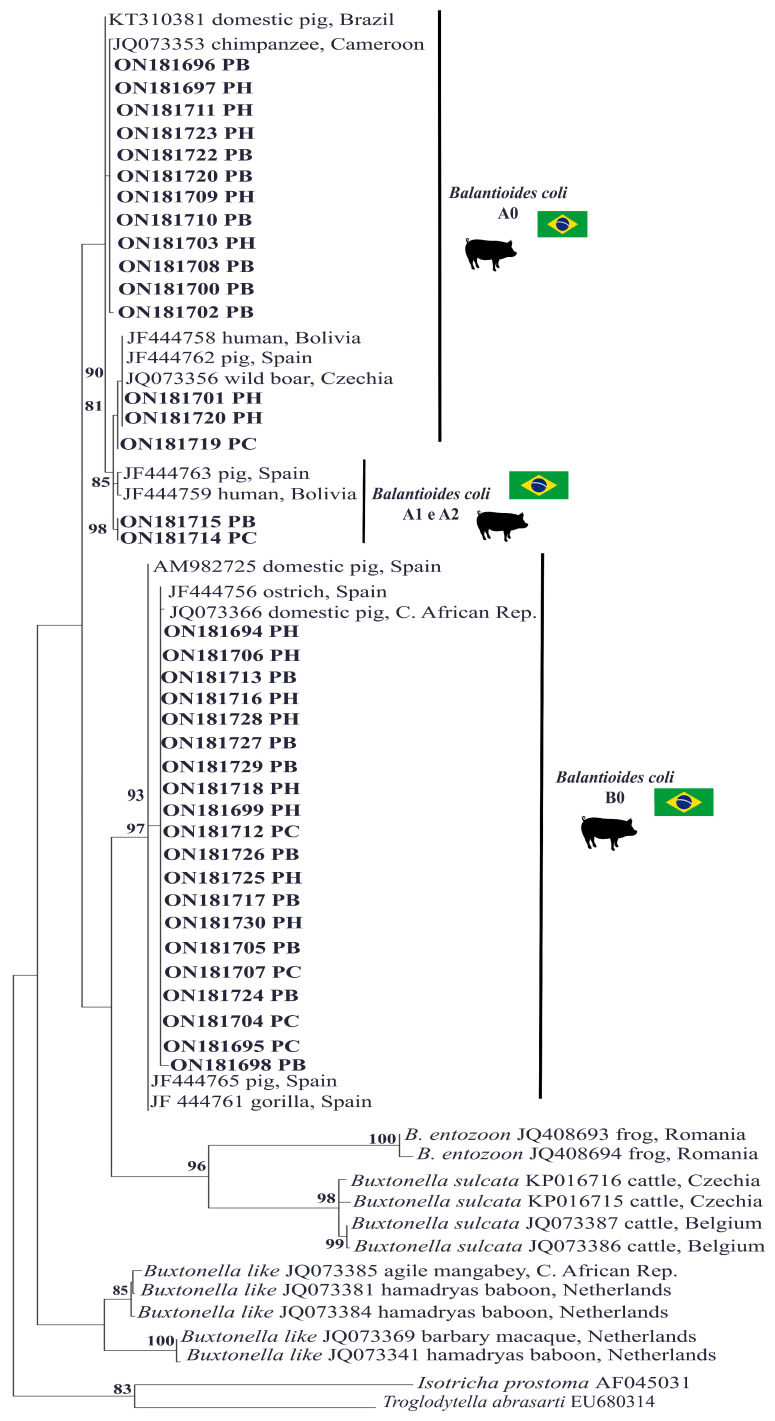
A phylogenetic tree was constructed based on the alignment of 370 base pairs (bp) of the DNA fragment of the rRNA gene and the ITS1-5.8S rRNA-ITS2 fragment from ciliated protozoa using the maximum likelihood method with the TIM2+F+ evolutionary Model G4. The sequences from this study are highlighted in bold and separated by the genetic variant. Sequences of *Isotricha prostoma* and *Troglodytella abrassarti* were used as outgroups. The numbers associated with the branches refer to the bootstrap values for 1000 replications.

All 37 sequences generated were compatible with *B. coli*, 15 of which were classified as genetic variant type A0, 2 as type A1/A2 and 20 as type B0. All of the sequences showed identity values greater than 97% when compared with reference sequences deposited in GenBank (Figure 2, Table 1). Furthermore, the sequences generated in this study were deposited in GenBank and received accession numbers ranging from ON181694 to ON181730. The analysis of the topography of the phylogenetic tree revealed that only the sequences ON181714 and ON181715 from the same fecal sample (isolate 78), isolated from the PC and PB culture media, respectively, were more closely grouped with the A1/A2 variants. The other sequences were included in the A0 and B0 variant groups (Figure 2).

Of the four samples that were successfully isolated in the three recommended culture media, two were found to have genetic variants whose molecular characterization differed according to the culture medium used for isolation; for example, sample 31 in the PC medium generated sequence ON181695, characterized as type B0; in the PB medium, this sample generated sequence ON181696, characterized as type A0; and in the PH medium, this sample generated sequence ON181697, characterized as type A0. Similarly, sample 78 in the PC medium generated sequence ON181714, characterized as type A1/A2; in the PB medium, this sample generated sequence ON181715, characterized as type A1/A2; and in the PH medium, this sample generated sequence ON181716, characterized as type B0 (Table 1 and Figure 2).

## 4. Discussion

In this study, the selection of pig fecal samples for in vitro isolation was prioritized by the number of forms compatible with *B. coli* visualized during direct examination to perform the qualitative comparison of the media supplemented with different sera and to increase the likelihood of successful isolation and subsequent maintenance, albeit in the short term. In general, for the isolation of intestinal protists, the use of fecal samples rich in parasitic cells is recommended, especially for those in which the culture technique is not a diagnostic tool [10]. This was also confirmed in the present study because even when beginning with fecal samples rich in forms of parasites, it was found during the analysis of the experimental results that, regardless of the serum added to the medium, not all the samples were successfully isolated and maintained until the 5th day of the incubation period, i.e., up to 120 h.

Successful protist isolation was achieved from the inoculated feces, in which trophozoites and/or cysts were previously visualized via light microscopy. No discrepancies were observed in the success of parasite isolation in the culture medium in relation to the parasite form that was inoculated. In general, the isolation of ciliates is usually achieved when a large number of mobile trophozoites are present in fecal samples, ruling out the need for the excystment stage [19]. Isolates obtained from feces containing mainly trophozoites have been reported in studies conducted in the Philippines, Brazil and India [11,19,26]. In most of the literature consulted, the form of the parasite that was inoculated into the culture medium was not reported [27,28,29,30,31,32]. This lack of information has made comparisons and the future replication of experimental isolation protocols difficult.

Several components of xenic media are essential for the isolation and maintenance of parasites. Among them, fetal bovine and horse sera, which are considered the main sources of nutrients, especially lipids, stand out. In general, for ciliated protists, especially for *B. coli*, serum is a key component of in vitro isolation and maintenance systems because without these elements, the protozoan population does not multiply, and the parasite is not isolated [33]. In many of the studies in which in vitro cultures of ciliated parasites morphologically compatible with *B. coli* were performed, horse serum was added to the culture medium [11,27,30,31,34]. However, there are still those who invested in fetal bovine serum [12,19,32].

It should be noted that the main drawback of whey production is the damage and stress to the animal that provides the biological material used as a basis for this input. In addition, serum of animal origin is one of the most expensive components of in vitro culture media, and its acquisition depends on the supply from the producer market or even through institutional partnerships. Thus, the efficiency of alternative methodologies that could replace this component should be assessed. Over the years, serum from coconut water has been tested for the isolation and/or maintenance of different microorganisms, such as poliovirus and parasites [15,16,17,18]. Despite being of Indian origin, coconuts have become well established in Brazil, providing a low-cost fruit. It contains liquid albumen (coconut water), which is a sterile, slightly acidic solution containing minerals, proteins, sugars, vitamins, fatty acids and growth factors (phytohormones) [13].

In this study, coconut water was analyzed qualitatively, and when compared to the other sera, coconut water also allowed the isolation of ciliated protozoans and the maintenance of most of the isolates for a short period, that is, until the 5th day of incubation, with 24 h intervals between successive subcultures. In addition, some isolates were maintained up to the 30th day of incubation, with 48 h intervals between subcultures, when fresh media was provided. This short-term maintenance was also evidenced in the isolation and in vitro maintenance of *Trypanosoma cruzi* and *Leishmania infantum* [15,16,17,18].

Despite the successful isolation and short-term maintenance of the protozoa in Pavlova media plus coconut water (PC), it was not possible to maintain the parasites for long periods in this system, as observed for Pavlova media plus fetal bovine serum (PB) and Pavlova with horse serum (PH). In general, the medium enriched with coconut water did not meet the nutritional needs of the protozoa and consequently could not sustain the subsequent subcultures. In the PC medium, as protozoan subcultures were performed, i.e., as the fecal residue was diluted via the introduction of fresh medium, there was also a decrease in the number of parasitic cells. Therefore, in the range of incubation periods of 1 to 2 days, a greater loss of isolates was recorded in the PC medium than in the other media since the first days are critical and decisive for the establishment of the isolate in vitro.

Although the PH medium presented similar results in terms of isolation frequency and maintenance as the PB medium, its main drawback was allowing the multiplication of other microorganisms, such as parabasilids, especially the vacuolar forms of *Blastocystis* sp., which multiply, hindering the subsequent maintenance of *B. coli*. This inconvenience was minimal in the PB media.

As evidenced in the photos of the microscope slides showing the sediments present in the culture tubes for each incubation time in the present study, after 96 to 120 h, the amount of fecal debris decreased significantly in the material analyzed. In addition, despite being a qualitative analysis, it was possible to visually verify that the number of parasitic cells increased markedly at these incubation times, especially in the PB and PH media.

Based on these characteristics, the isolates in cultivation were subjected to DNA extraction and molecular analysis after the fifth day of incubation. Therefore, in this study, DNA extraction did not need to be carried out directly from feces. It is worth noting that the polymerase chain reaction carried out directly from fecal material is more difficult to amplify and can generate very noisy electropherograms. This problem of analyzing DNA directly extracted from fecal material has already been reported in studies that investigated ciliates in the feces of guinea pigs and in the feces of pigs and nonhuman primates with primers that amplify regions that evolve relatively quickly, such as ITS1 and ITS2 [9,33]. The molecular characterization of cultivated isolates was performed in this study to verify the potential of the media to generate biological material for molecular identification, minimizing the number of PCR inhibitors, and increasing the chance of the amplification of target protozoan DNA by means of cell abundance, generating reliable and easy-to-interpret electropherograms. In addition, molecular characterization was used to taxonomically identify the species of ciliate protozoan and the genetic variants circulating in the study location.

The isolates that were successfully obtained and maintained until the fifth day of incubation in all three culture media (PC, PB and PH) were subjected to molecular analysis. This analysis revealed that the PC medium generated the lowest number of amplified products via PCR. A large portion of the positive-PCR products were generated from protozoa maintained in the PB and PH media. This result was expected because although mobile cells were visualized on microscope slides in the PC medium, they did not appear to be present in sufficient quantities to generate amplifiable DNA during PCR. Although a quantitative analysis was not performed, the low number of parasitic cells in the PC medium used in this study was also observed in the photodocumentation generated in this medium; therefore, this is not the most appropriate culture system for generating parasitic cells for use in molecular studies.

A total of 37 nucleotide sequences were generated from all 19 samples and were molecularly characterized as *B. coli*. These strains presented high values of identity when compared with sequences of *B. coli* isolated and maintained in xenic culture from pig feces collected at a property located in Brazil; from feces of a captive chimpanzee in Cameroon; from a human patient in Bolivia; from a wild boar and captive pigs in the Czech Republic and Central African Republic, respectively; and from captive pigs, ostriches and gorillas in Spain [6,8,9,22]. Although pigs are considered the main reservoir of *B. coli*, the taxonomic confirmation of the parasite can only be performed using molecular tools, since the Ciliophora Group has a large number of species that may be of environmental origin or also colonize the gastrointestinal tract of mammals; in these cases, there are species that are morphologically similar to *B. coli*, such as *Buxtonella* sp. [4,6,35]. Furthermore, it was also possible to verify the profile of the parasite’s genetic variants isolated with culture media plus different sources of serum. Among the variants characterized in the nucleotide sequences of *B. coli* obtained from the isolates, the A0, A1/A2 and B0 types were found. However, most of the sequences exhibited a B0-type variant pattern. This variant was present in isolates generated from fecal samples collected at almost all the sampled family farms, except for G and H.

The B0 variant was also responsible for the largest number of cases observed in molecular epidemiology studies of *B. coli* obtained from the feces of domestic pigs in China and Korea [7,36]. In addition to type B0, type A, especially A0, was observed in isolates from pigs from farms A, D, E, G and H. It is important to note that this variant has been identified in the feces of humans and other animal species, such as guinea pigs, pigs, ostriches and nonhuman primates [8].

Despite the inference regarding the zoonotic character of the *B. coli* A0 variant, there are still few nucleotide sequences of the parasite originating from human biological material deposited in specific storage banks of molecular information. This lack of data emphasizes the need for additional studies, especially in countries with the highest epidemiological indices of human balantiosis, as is the case in Brazil, for which a systematic review has already been conducted [4].

After the sequences were phylogenetically analyzed, only two were found to be clustered closer to the A1/A2 cluster than to the A0 type. The A1/A2 variants have rarely been reported in the literature. After analyzing the sequences in detail, it was found that the ITS2 region was the most important region for characterizing the sequences closer to the A1/A2 cluster (especially the A1 type). Similar findings have been reported for pigs in Spain as well as other animal species, including guinea pigs [8,33].

In the molecular characterization of the *B. coli* genetic variants of the isolates, no mixed nucleotide sequences were observed; that is, electropherograms containing double peaks indicating the concomitant amplification of DNA from variants A and B or even from subvariants were not generated. However, the presence of populations with distinct genetic variants, i.e., type A or B, was evidenced from the molecular characterization of the isolates generated from both fecal samples “isolate 31 and 78” when the isolates generated from the different culture media were analyzed. It is important to highlight that with *B. coli*, in its genome there are at least two rRNA genes (one that encodes type A rRNA sequences and the other as type B) and they are in different locations [8]. In this way, the establishment of different genetic variants of *B. coli* according to the medium used may be related to the unequal distribution of parasitic cells during the production of inoculum between the culture media or even selection for a population of cells that was expressing certain type-specific variant sequences.

Unlike these results, the A0 type variant sequence was the only one characterized in *B. coli* isolates cultured in PB medium from pig and Cynomolgus monkey feces also raised on farms in the state of Rio de Janeiro, Brazil [9]. The greater diversity of genetic variants observed in the *B. coli* isolates obtained in the present study may be related to the early molecular analysis that was performed during the first days of in vitro parasite maintenance, unlike the aforementioned study in which molecular characterization was performed on isolates that had been maintained in culture for a long period. However, more studies are needed to further corroborate these findings.

## 5. Conclusions

Based on these analyses, it was possible to verify that Pavlova medium plus coconut water allowed the isolation and maintenance of *B. coli* for a short period of time; this is the first study in which coconut water was evaluated as an alternative source to replace serum of animal origin. However, Pavlova plus coconut water was not an appropriate medium for use in studies involving the molecular characterization and epidemiology of the parasite or for long-term maintenance. Thus, this medium requires additional analyses, including quantitative analyses and other evaluations at different concentrations, to support its improvement. The results of this study also indicate that the type of serum added to the cultures may have influenced the establishment of the *B. coli* variant type and, consequently, the molecular characterization of the sample; further studies are needed to corroborate these results.

## Figures and Tables

**Table 1:** Information on the diagnosis, in vitro culture and molecular analysis of the successfully isolated protozoa.

Sample	Farms	Form Detected	Medium	PCR Result	Sequencing	GenBank	Variant	Identity
17	A	Trophozoite	PC +	PCR −	N	-	-	-
17	A	Trophozoite	PB +	PCR −	N	-	-	-
17	A	Trophozoite	PH +	PCR +	*Balantioides coli*	ON181694	B0	98.9–100%
31	A	Trophozoite	PC +	PCR +	*Balantioides coli*	ON181695	B0	98.9–100%
31	A	Trophozoite	PB +	PCR +	*Balantioides coli*	ON181696	A0	97.8–99.4%
31	A	Trophozoite	PH +	PCR +	*Balantioides coli*	ON181697	A0	98.1–99.4%
34	B	Trophozoite	PC −	N	N	-	-	-
34	B	Trophozoite	PB +	PCR +	*Balantioides coli*	ON181698	B0	98–98.9%
34	B	Trophozoite	PH −	N	N	-	-	-
38	C	Trophozoite	PC −	N	N	-	-	-
38	C	Trophozoite	PB +	PCR −	N	-	-	-
38	C	Trophozoite	PH +	PCR +	*Balantioides coli*	ON181699	B0	98.9–99.1%
44	D	Trophozoite/cyst	PC −	PCR −	-	-	-	-
44	D	Trophozoite/cyst	PB +	PCR +	*Balantioides coli*	ON181700	A0	98.1–99.4%
44	D	Trophozoite/cyst	PH +	PCR +	*Balantioides coli*	ON181701	A0	98.3–100%
53	D	Trophozoite	PC −	N	N	-	-	-
53	D	Trophozoite	PB +	PCR +	*Balantioides coli*	ON181702	A0	98.3–99.1%
53	D	Trophozoite	PH +	PCR +	*Balantioides coli*	ON181703	A0	98.1–99.4%
67	D	Trophozoite	PC +	PCR +	*Balantioides coli*	ON181704	B0	98.9–100%
67	D	Trophozoite	PB +	PCR +	*Balantioides coli*	ON181705	B0	98.9–100%
67	D	Trophozoite	PH +	PCR +	*Balantioides coli*	ON181706	B0	98.9–100%
68	E	Trophozoite/cyst	PC +	PCR +	*Balantioides coli*	ON181707	B0	98.9–100%
68	E	Trophozoite/cyst	PB +	N	N	-	-	-
68	E	Trophozoite/cyst	PH +	N	N	-	-	-
69	E	Cyst	PC −	N	N	-	-	-
69	E	Cyst	PB +	PCR +	*Balantioides coli*	ON181708	A0	98.1–99.4%
69	E	Cyst	PH +	PCR +	*Balantioides coli*	ON181709	A0	98.1–99.4%
75	E	Cyst	PC +	PCR −	N	-	-	-
75	E	Cyst	PB +	PCR +	*Balantioides coli*	ON181710	A0	98.1–99.4%
75	E	Cyst	PH +	PCR +	*Balantioides coli*	ON181711	A0	98.1–99.4%
76	A	Trophozoite/cyst	PC +	PCR +	*Balantioides coli*	ON181712	B0	98.9–100%
76	A	Trophozoite/cyst	PB +	PCR +	*Balantioides coli*	ON181713	B0	98.9–100%
76	A	Trophozoite/cyst	PH +	PCR −	N	-	-	-
78	F	Trophozoite/cyst	PC +	PCR +	*Balantioides coli*	ON181714	A1/A2	97.8–99.1%
78	F	Trophozoite/cyst	PB +	PCR +	*Balantioides coli*	ON181715	A1/A2	97.8–99.1%
78	F	Trophozoite/cyst	PH +	PCR +	*Balantioides coli*	ON181716	B0	98.9–100%
79	F	Trophozoite/cyst	PC +	PCR −	N	-	-	-
79	F	Trophozoite/cyst	PB +	PCR +	*Balantioides coli*	ON181717	B0	98.9–100%
79	F	Trophozoite/cyst	PH +	PCR +	*Balantioides coli*	ON181718	B0	98.9–100%
85	G	Cyst	PC +	PCR +	*Balantioides coli*	ON181719	A0	98.6–99.7%
85	G	Cyst	PB +	PCR +	*Balantioides coli*	ON181720	A0	98.1–99.4%
85	G	Cyst	PH +	PCR +	*Balantioides coli*	ON181721	A0	98.3–100%
107	H	Cyst	PC −	N	N	-	-	-
107	H	Cyst	PB +	PCR +	*Balantioides coli*	ON181722	A0	98.1–99.4%
107	H	Cyst	PH +	PCR +	*Balantioides coli*	ON181723	A0	98.1–99.4%
137	I	Cyst	PC +	PCR −	N	-	-	-
137	I	Cyst	PB +	PCR +	*Balantioides coli*	ON181724	B0	98.9–100%
137	I	Cyst	PH +	PCR +	*Balantioides coli*	ON181725	B0	98.9–100%
148	I	Cyst	PC −	N	N	-	-	-
148	I	Cyst	PB +	PCR +	*Balantioides coli*	ON181726	B0	98.9–100%
148	I	Cyst	PH +	PCR −	N	-	-	-
177	J	Trophozoite/cyst	PC −	N	N	-	-	-
177	J	Trophozoite/cyst	PB +	PCR +	*Balantioides coli*	ON181727	B0	98.9–100%
177	J	Trophozoite/cyst	PH +	PCR +	*Balantioides coli*	ON181728	B0	98.9–100%
180	J	Trophozoite/cyst	PC +	PCR −	N	-	-	-
180	J	Trophozoite/cyst	PB +	PCR +	*Balantioides coli*	ON181729	B0	98.9–100%
180	J	Trophozoite/cyst	PH +	PCR +	*Balantioides coli*	ON181730	B0	98.9–100%

A to J: indicators used to describe the family farms on which the pigs were raised; PC: Pavlova with coconut water; PB: Pavlova with fetal bovine serum; PH: Pavlova with equine serum; N: result not obtained; −: not applicable.

**Table 2:** Frequency of ciliated protozoan isolates obtained from pig fecal samples according to viability duration (in days) in modified Pavlova culture media plus coconut water (PC), fetal bovine serum (PB) and horse serum (PH).

Duration (in Days) of Parasite Viability In Vitro	PC	PB	PH	*p* Value
0–1	12 (38.7%)	10 (32.2%)	11 (35.5%)	0.8686
1–2	3 (9.7%)	0	0	0.0450 *
2–3	1 (3.2%)	0	0	0.3639
3–4	4 (12.9%)	2 (6.4%)	2 (6.4%)	0.5786
4–5	2 (6.4%)	1 (3.2%)	1 (3.2%)	0.7701
5–30	9 (29%)	14 (45.2%)	12 (38.7%)	0.4188
30–60	0	2 (6.4%)	2 (6.4%)	0.3517
60–90	0	0	1 (3.2%)	0.3639
90–120	0	0	1 (3.2%)	0.3639
120 or more	0	2 (6.4%)	1 (3.2%)	0.3558
Total	31	31	31	

* *p* value ≤ 0.05 is considered statistically significant. The gray part of the table shows the parasites maintained until the 5th day of incubation in the culture medium.

## Data Availability

The original contributions presented in the study are included in the article.
